# Hypoxia induced changes in miRNAs and their target mRNAs in extracellular vesicles of esophageal squamous cancer cells

**DOI:** 10.1111/1759-7714.13295

**Published:** 2020-01-10

**Authors:** Fangyu Chen, Li Chu, Jie Li, Yu Shi, Bing Xu, Junjie Gu, Xijuan Yao, Meng Tian, Xi Yang, Xinchen Sun

**Affiliations:** ^1^ The First School of Clinical Medicine Nanjing Medical University Nanjing China; ^2^ Department of Radiation Oncology The First Affiliated Hospital of Nanjing Medical University Nanjing China; ^3^ Department of Radiation Oncology Fudan University Shanghai Cancer Center Shanghai China; ^4^ Department of Oncology, Shanghai Medical College Fudan University Shanghai China; ^5^ Department of Radiotherapy Affiliated Hospital of Nantong University Nantong China

**Keywords:** Esophageal cancer, extracellular vesicles, hypoxia, microRNA, tumor microenvironment

## Abstract

**Background:**

Extracellular vesicles (EVs) are endogenous membrane vesicles with a diameter of 30–200 nm. It has been reported that hypoxic cancer cells can release numerous EVs to mediate multiple regional and systemic effects in the tumor microenvironment.

**Methods:**

In this study, we used ultracentrifugation to extract EVs secreted by TE‐13, an esophageal squamous carcinoma (ESCC) cell line during normoxia and hypoxia and performed high‐throughput sequencing to detect exosomal miRNAs. Gene ontology (GO) and KEGG pathway analyses were used to reveal pathways potentially regulated by the miRNAs.

**Results:**

A total of 10 810 miRNAs were detected; 50 were significantly upregulated and 34 were significantly downregulated under hypoxic environment. GO analysis identified enrichment of protein binding, regulation of transcription (DNA‐templated), and membrane as molecular function, biological process, and cellular component, respectively. KEGG pathway analysis revealed cancer‐associated pathways, phospholipase D signaling pathway, autophagy, focal adhesion and AGE‐RAGE signaling as the key pathways. Further verification experiment from qRT‐PCR indicated that miR‐128‐3p, miR‐140‐3p, miR‐340‐5p, miR‐452‐5p, miR‐769‐5p and miR‐1304‐p5 were significantly upregulated in EVs from hypoxia TE‐13 cells while miR‐340‐5p was significantly upregulated in two other ESCC cells, ECA109 and TE‐1.

**Conclusion:**

This study, for the first time reveals changes in the expression of exosomal miRNAs in hypoxic ESCC cells and these findings will act as a resource to study the hypoxic tumor microenvironment and ESCC EVs.

## Introduction

Esophageal cancer is the sixth leading cause of cancer‐related death worldwide. A total of 455,00 esophageal cancer diagnoses and 400, 200 deaths were reported in 2012.^1^ The two main types of esophageal cancer are squamous cell carcinoma and adenocarcinoma.^2^ Although multimodal treatment approaches including surgery, radiotherapy, and chemotherapy have been applied, the five‐year survival rate for esophageal squamous cell carcinoma (ESCC) remains less than 30%.^3^, ^4^, ^5^ Radiotherapy is an essential therapy for patients with ESCC. However, a large portion of ESCC tumors develop resistance to radiotherapy, indicating the importance of enhancing the radiation sensitivity of ESCC.

Early studies have found that the radiation sensitivity of cancer cells is largely determined by the presence of oxygen. For most cells, the oxygen enhancement ratio is 2.5–3.0 after a single dose of radiation. This means that the radiation dose required to kill hypoxic cells is 2.5 to three times more than the dose required to kill aerobic cells.^6^ The tumor microenvironment (TME) is made up of different cell types, extracellular matrix, and numerous extracellular molecules.^7^ Accumulated evidence underscores the role of TME in promoting tumor formation and progression.^8^, ^9^ Hypoxia is a phenomenon commonly observed in the TME and is defined as the reduction in tissue oxygen tension.^9^, ^10^ Several cancer characteristics, such as neovascularization, alteration of energy metabolism, immune escape, initiation of invasion and metastasis, uncontrolled inflammation, sustaining proliferation, repressing apoptosis, and genomic instability are associated with hypoxia.^11^, ^12^, ^13^, ^14^, ^15^, ^16^

Extracellular vesicles (EVs) are endogenous membrane vesicles with a diameter of 30–200 nm and contain proteins, lipids, and various types of nucleic acids, including DNA and RNA. Intercellular communication in the tumor microenvironment is suggested to be involved in therapeutic resistance. Tumor‐derived EVs contain proteins and nucleic acids, which serve as key mediators of cellular communication, promoting tumor progression and treatment resistance.^17^ Hypoxic cells release numerous EVs which have been shown to mediate multiple regional and systemic effects, such as local neovascularization and invasiveness as well as distant organ metastasis.^18^ MicroRNAs (miRNAs) are non‐coding small RNA molecules that regulate gene expression at the post‐transcriptional/translational level. They play a considerable role not only in normal development, but also in human diseases, including malignancy.^19^ Exosomal miRNAs in the blood appear stable and have gained potential as noninvasive biomarkers in the context of tumor hypoxia, which regulates miRNA transcription and maturation.^20^ Several studies have investigated exosomal miRNAs as tumor biomarkers.^21^, ^22^, ^23^, ^24^, ^25^ In this context, we hypothesized that exosomal miRNAs might reflect hypoxic tumor components and consequently, imply response to nCRT treatment in patients with ESCC. Here, we isolated EVs from ESCC cell lines and characterized specific exosomal miRNA constituents by high‐throughput sequencing.

## Methods

### Cell culture and hypoxic treatment

The ESCC cell line TE‐13, ECA109, TE‐1 was purchased from a typical cell culture collection committee of the Chinese Academy of Sciences Library. The cells were cultured in RPMI‐1640 medium (Gibco, CA, USA) supplemented with 10% FBS (Gibco, CA, USA), 100 U/mL penicillin and 100 μg/mL streptomycin at 37°C in a humidified atmosphere of 5% CO_2_. Cells were routinely examined for *Mycoplasma* contamination. To induce hypoxic conditions (<1% O_2_), cells were cultured at 37°C in the same incubator in an AnaeroPack jar with AneroPack‐Anaero (Mitsubishi, Tokyo, Japan) according to the manufacturer's instructions. The hypoxic environment was confirmed by hypoxia inducible factor 1 subunit alpha (HIF‐1α) expression.

### EV isolation and identification

FBS was depleted of EVs by ultracentrifugation at 140000 *g* at 4°C for 16 hours (Beckman Coulter Avanti J‐30I, CA, USA), and the supernatant was collected and filtered using a 0.22 μm filter (Millipore, MA, USA). Before EV isolation, the cells were cultured in normal medium until 50% confluency and then the medium was replaced with RPMI‐1640 with 10% EV‐depleted FBS and cultured under normoxic or hypoxic conditions. After 48 hours, cell culture medium was collected (50 mL), and EVs were isolated by differential centrifugation at 500 *g* for five minutes, 2000 *g* for 15 minutes, and 12 000 *g* for 30 minutes, to remove floating cells and cellular debris. The supernatant was then ultracentrifuged at 120000 *g* for 70 minutes and the pellet resuspended in PBS and ultracentrifuged. The EVs were used immediately for further experiments.

EVs were resuspended in PBS and fixed with 4% paraformaldehyde and 4% glutaraldehyde in 0.1 M phosphate buffer (pH 7.4) and kept at 4°C until analysis. A drop of EV sample from each condition (normoxic or hypoxic) was placed on a carbon‐coated copper grid and immersed in 2% phosphotungstic acid solution (pH 7.0) for 30 seconds. The preparation was examined under an electron microscope (JEM‐1200EX, JEOL Ltd., Tokyo, Japan) at an acceleration voltage of 80 kV. The size distribution and concentration of EVs were analyzed by nanoparticle tracking analysis (NTA) using a ZetaView particle tracker from ParticleMetrix (Meerbusch, Germany). CD63 (1:1000, ab68418, Abcam, MA, USA) and CD81 (1:1000, ab155760, Abcam, MA, USA) were used as exosomal markers and Calnexin (1:1000, ab75801, Abcam, USA) was used as a negative control for EVs.

### Western blotting

Samples of cells, supernatant and EVs were washed and resuspended in RIPA lysis buffer (Beyotime, Shanghai, China) with protease inhibitor mixture (Millipore, MA, USA). Proteins were separated based on their molecular weight by sodium dodecyl sulphate polyacrylamide gel electrophoresis and then transferred onto a polyvinylidene fluoride membrane (Millipore, MA, USA). The membranes were blocked with 5% skim milk powder in Tris‐buffered saline containing Tween 20 (TBST) for two hours, and the membranes were then incubated at 4°C overnight with specific primary antibodies. The membranes were rinsed in TBST for three times (10 minutes each time), and incubated in secondary antibodies at room temperature for two hours and were then washed again in TBST (three times, 10 minutes each time). Protein expression levels were detected by ECL Plus (Millipore, MA, USA) using a Bio‐Imaging System.

### Construction of small RNA libraries and bioinformatic analysis

Raw reads were analyzed using an in‐house program, ACGT101‐miR (LC Sciences, Texas, USA) to remove adapter dimers, junk, low complexity, common RNA families (rRNA, tRNA, snRNA, snoRNA), and repeats. Subsequently, unique sequences with a length of approximately 18–26 nucleotides were mapped to specific species precursor miRNAs in miRBase 22.0 by BLAST search to identify known miRNAs and novel 3p‐ and 5p‐ derived miRNAs. Length variation at both 3′ and 5′ ends and one mismatch within the sequence was allowed in the alignment. The unique sequences mapping to specific species mature miRNAs in hairpin arms were identified as known miRNAs. The unique sequences mapping to the other arm of known specific species precursor hairpin opposite to the annotated mature miRNA‐containing arm were considered novel 5p‐ or 3p‐derived miRNA candidates. The remaining sequences were mapped to other selected species' precursor miRNA (with the exclusion of specific species) in miRBase 22.0 by BLAST search. The mapped pre‐miRNAs were further BLASTed against the genomes of specific species to determine their genomic locations. We defined the above two as known miRNAs. The unmapped sequences were BLASTed against the specific genomes, and the hairpin RNA structure containing sequences were predicted from the flank 80 nucleotide sequences using RNAfold software (http://rna.tbi.univie.ac. at/cgi‐bin/RNAfold.cgi). The criteria for secondary structure prediction were:^1^ number of nucleotides in one bulge in stem (≤12) 2 number of base pairs in the stem region of the predicted hairpin (≥16) 3 cutoff of free energy (kCal/mol ≤−15) 4 length of hairpin (top and bottom stems + terminal loop ≥50) 5 length of hairpin loop (≤20) 6 number of nucleotides in one bulge in mature region (≤8) 7 number of biased errors in one bulge in mature region (≤4) 8 number of biased bulges in mature region (≤2)^9^ number of errors in mature region (≤ 7) 10 number of base pairs in the mature region of the predicted hairpin (≥ 12) 11 percent of mature in stem (≥80).

### Analysis of differentially expressed miRNAs

Differential expression of miRNAs based on normalized deep‐sequencing counts was analyzed by selectively using Fisher exact test, Chi‐squared 2 x 2 test, Chi‐squared n x n test, Student's *t*‐test, or ANOVA, based on the experimental design. The significance threshold was set at 0.05 in each test.

### Prediction of target genes of miRNAs

To predict the genes targeted by most abundant miRNAs, two computational target prediction algorithms (TargetScan 50 and Miranda 3.3a) were used to identify miRNA binding sites. Finally, the data predicted by both algorithms were combined and the overlaps were calculated. The GO terms and KEGG pathways of the most abundant miRNAs and miRNA targets were also annotated.

### miRNA RT‐PCR

Following the manufacturer's instructions, total RNA was extracted with TRIzol reagent (Invitrogen, USA). For miRNA RT‐PCR, we used a Hairpin‐it miRNA qPCR Quantitation Kit (GenePharma, China) to perform Target‐specific reverse transcription and the TaqMan microRNA assay. The reactions were processed using a Realtime PCR System (Applied Biosystems 7500, Carlsbad, CA, USA) with SYBR Premix Ex Taq Kit (TaKaRa, Japan). The expression level was normalized to external controls cel‐miR‐39 (RiboBio, Guangzhou, China). Results were shown in form of relative expression calculated by the 2−ΔΔCT method.

### Statistical analysis

Statistical analysis was performed by two‐way ANOVA using GraphPad Prism 8.0 software (San Diego, CA) and *P* < 0.05 was statistically significant.

## Results

### Characterization of normoxic and hypoxic ESCC EVs

**Figure 1:**
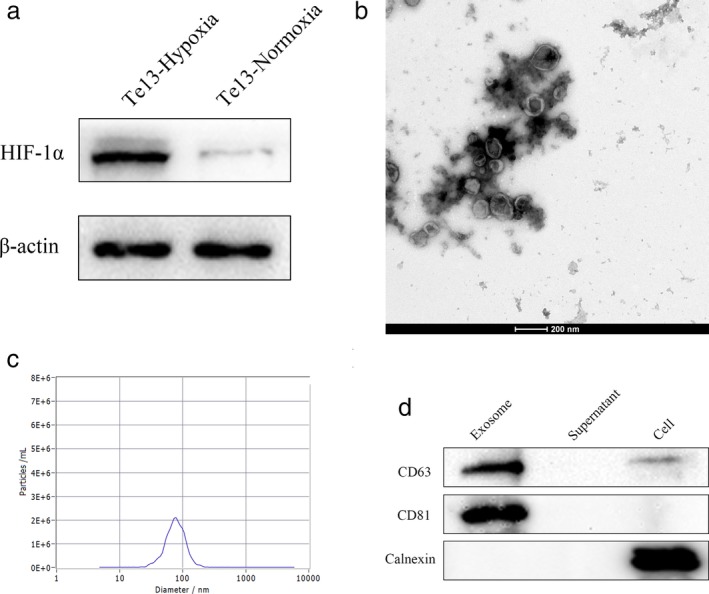
Confirmation of hypoxia and identification of EVs from TE‐13 cells. (**a**) Hypoxia environment was confirmed by HIF‐1α expression; (**b**) EVs were defined by a lipid bilayer under TEM; (**c**) NTA analysis showing the mean vesicle size distribution of 74.1 nm; (**d**) Western blot of EVs showing enriched expression of exosomal marker CD63 and CD81 and no expression of endoplasmic reticulum protein Calnexin.

Following culture under normoxic and hypoxic conditions for 48 hours, features of isolated EVs were characterized in detail (Fig. 1). First, hypoxic responses were confirmed by expression of HIF‐1α in hypoxic cells (Fig. 1(a)). Next, transmission electron microscopy (TEM) revealed that the isolated EVs were defined by a lipid bilayer (Fig. 1(b)), and further measurements by NTA showed a mean vesicle size of 74.1 nm (Fig. 1(c)). Finally, released vesicles from both normoxic and hypoxic TE13 cells expressed proteins known to be enriched in EVs (CD63 and CD81) and showed no expression of the endoplasmic reticulum marker (Calnexin) used as a negative control, as observed by western blotting analysis (Fig. 1(d)).

### Differentially expressed miRNAs in EVs

**Figure 2:**
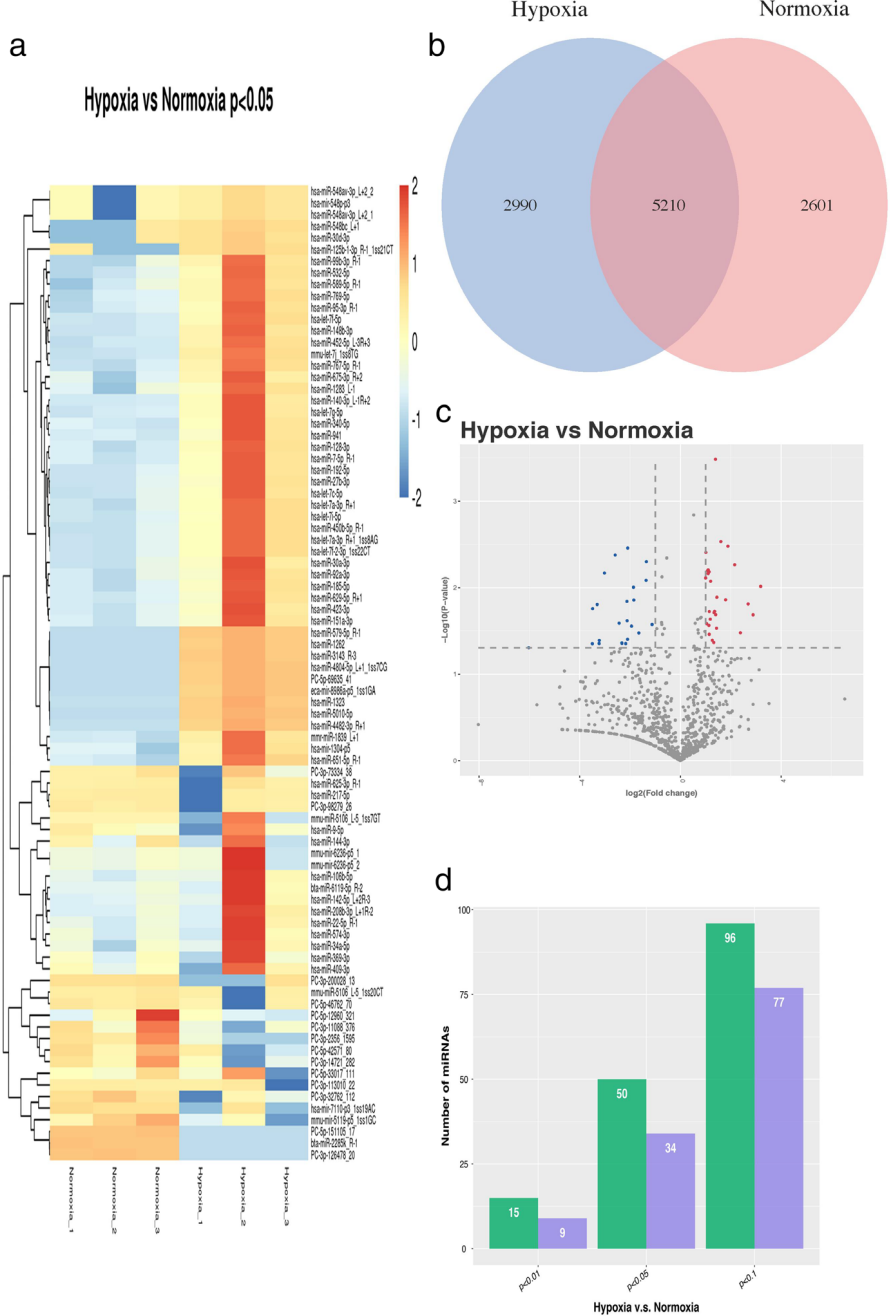
Differentially expressed miRNAs in hypoxic and normoxic EVs. (**a**) Heat map showing clustering of changed miRNAs. Red represents upregulated miRNAs and blue represents downregulated miRNAs; (**b**) Venn diagram showing the number of overlapped miRNAs in each group; (**c**) Volcano plot revealing altered miRNAs in hypoxic and normoxic EVs; (**d**) Bar plot indicating number of altered miRNAs after hypoxia treatment at different *P*‐values. 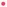 up, 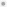 no_diff, 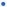 down, 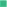 up‐regulated, 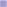 down‐regulated.

A total of 10 810 miRNAs were detected in EVs from the two groups. Only 2601 miRNAs were detected in the normoxic group and 2990 miRNAs in the hypoxic group and 5210 were detected in both groups (Fig. 2). Of the total 10 810 miRNAs, 50 were significantly upregulated and 34 were significantly downregulated after hypoxic treatment when compared to those in controls (*P* < 0.05, fold change ≥2) (Table 1, Table S1).

**Table 1:** Significantly altered miRNAs in hypoxic EVs (*P* < 0.01) *inf: infinity

miRNA	Regulation	Fold change	*P‐*value
hsa‐miR‐1262	up	inf*	0.000869564
hsa‐miR‐769‐5p	up	3.06	0.002934105
hsa‐mir‐1304‐p5	up	3.71	0.00333358
PC‐3p‐73334_38	down	0.23	0.003489675
hsa‐miR‐128‐3p	up	2.02	0.003914834
PC‐3p‐2356_1595	down	0.17	0.004213607
hsa‐miR‐369‐3p	down	0.39	0.005009377
mmu‐let‐7j	up	4.47	0.005458252
hsa‐miR‐95‐3p	up	2.15	0.0062081
hsa‐miR‐532‐5p	up	2.11	0.006414664
hsa‐miR‐140‐3p	up	2.21	0.006570997
PC‐3p‐200028_13	down	0.12	0.006795539
hsa‐miR‐192‐5p	up	2.15	0.006911668
hsa‐miR‐3143	up	inf	0.006983654
hsa‐miR‐340‐5p	up	2.01	0.007744756
PC‐5p‐151105_17	down	‐inf	0.008038862
hsa‐miR‐409‐3p	down	0.39	0.008233985
hsa‐miR‐452‐5p	up	2.31	0.008441793
hsa‐miR‐144‐3p	down	0.28	0.009919166
hsa‐miR‐125b‐1‐3p	up	9.15	0.00968456

### Functional analysis of miRNA target genes

**Figure 3:**
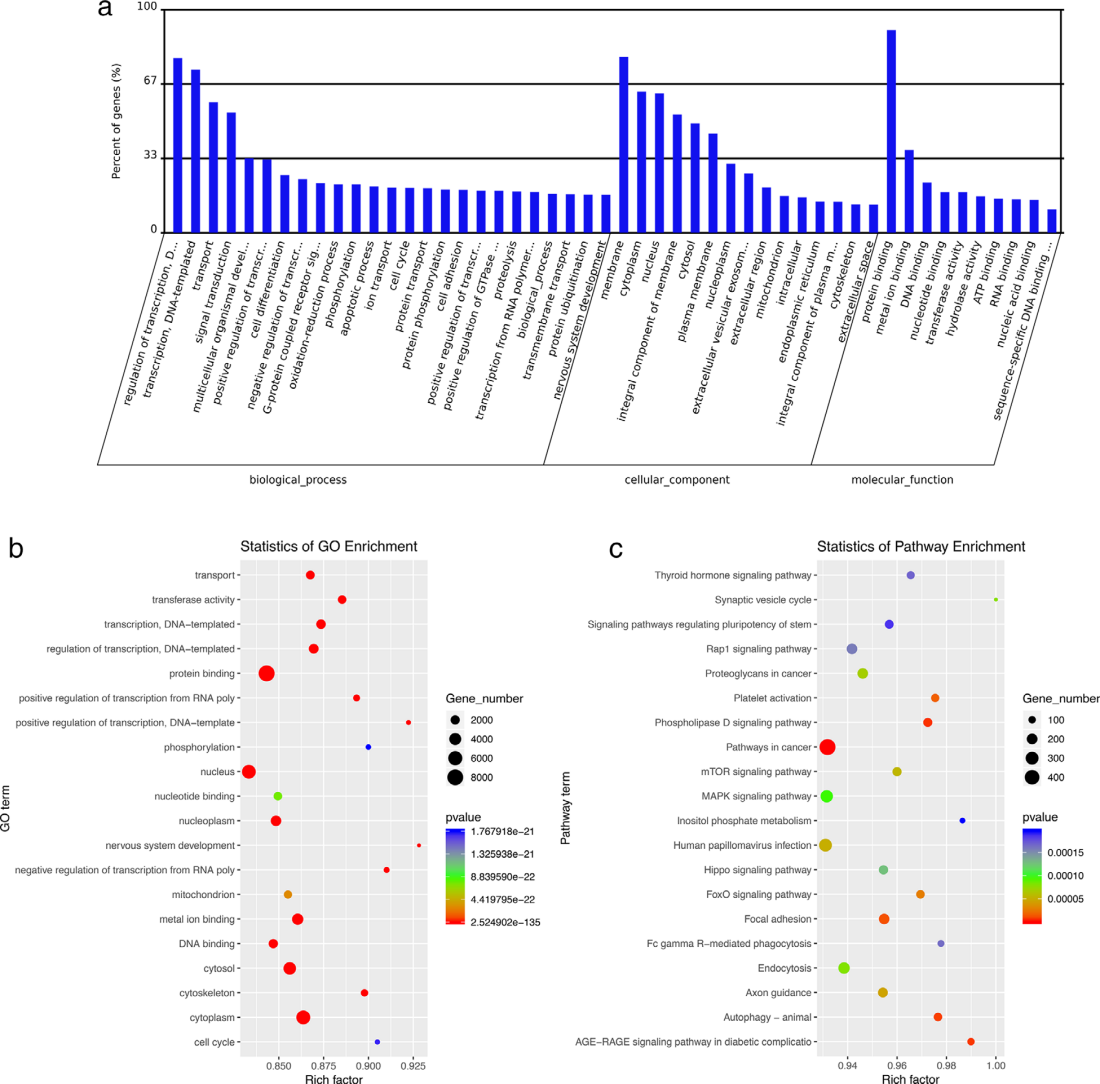
GO and KEGG analysis of differentially expressed miRNAs and target mRNAs. (**a**) Bar plot revealing GO analysis of molecular function, biological process and cellular component; (**b**) Scatter plot of GO enrichment analysis; (**c**) Scatter plot of KEGG signaling pathway analysis. Rich factor = the percentage of enriched genes in the total number of the genes in the pathway.

We next performed a GO analysis to better understand the functional association of target genes with the differentially expressed miRNAs (Fig. 3(a) and (b)). Our GO analysis included three parts: molecular function (MF), biological process (BP) and cellular component (CC). We determined two main functions for the first part (MF): protein binding and metal ion binding. Their function with respect to BP was determined to be regulation of transcription (DNA‐templated). When classified according to CC, membrane was identified as the largest component.

**Figure 4:**
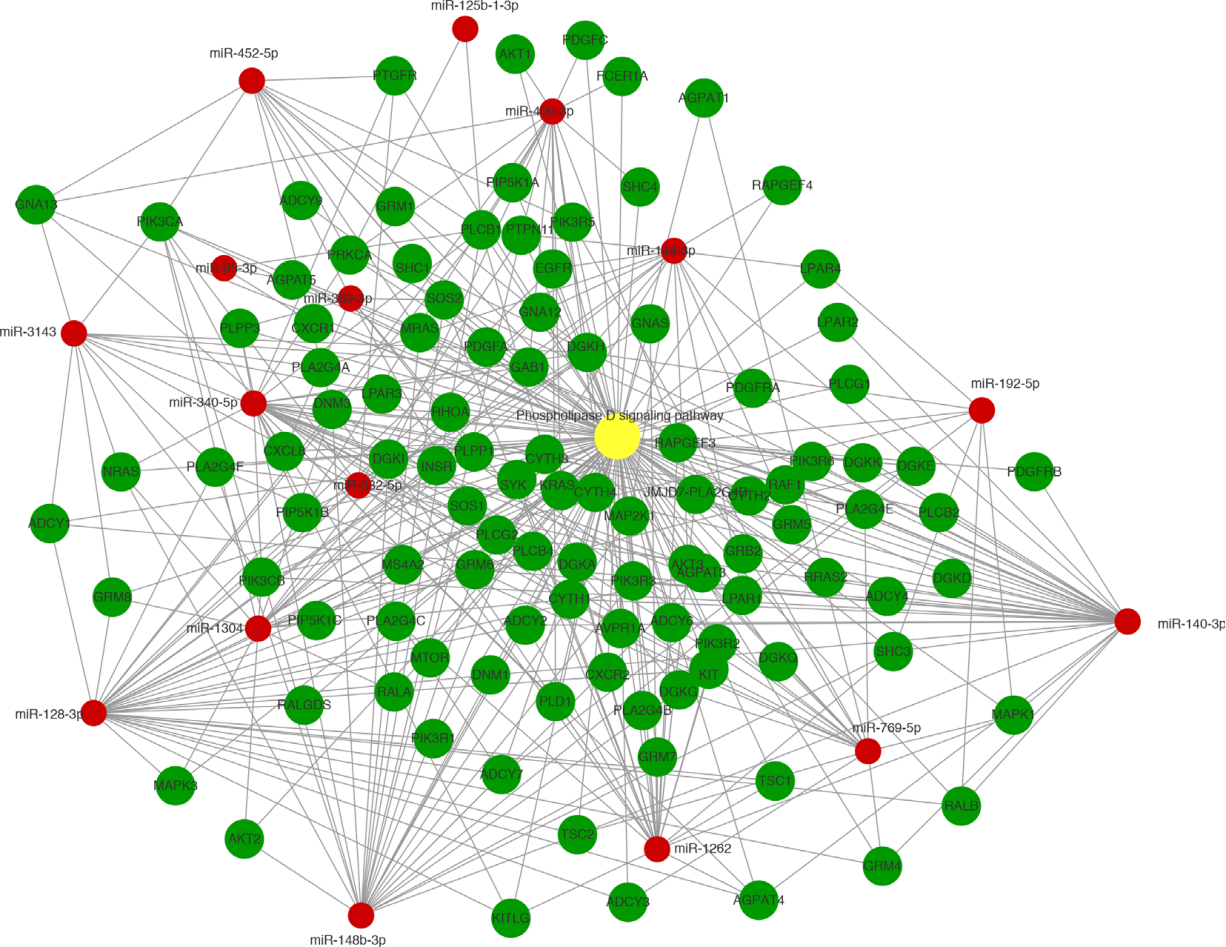
Analysis of miRNA‐mRNA regulatory network concerning Phospholipase D signaling pathway. 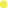 Phospholipase D signaling pathway, 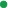 gene, 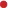 miRNA.

Next, the function of the predicted target mRNAs of the identified miRNAs was analyzed using KEGG pathway analysis (Fig. 3(c), Table 2, Table S2). Differentially expressed miRNAs and their target mRNAs were determined to be mostly involved in cancer‐related pathways, phospholipase D signaling pathway (Fig. 4) and some other pathways such as focal adhesion and autophagy. AGE‐RAGE with high enrichment factor may be also involved.

**Table 2:** KEGG pathways affected by altered exosomal miRNAs (*P* < 0.001)

KEGG pathway	gene number	*P‐*value
Pathways in cancer	492	1.03E‐07
Phospholipase D signaling pathway	141	4.88E‐06
AGE‐RAGE signaling pathway in diabetic complications	98	5.90E‐06
Autophagy ‐ animal	125	7.36E‐06
Focal adhesion	190	1.16E‐05
Platelet activation	119	1.59E‐05
FoxO signaling pathway	127	2.76E‐05
Axon guidance	167	4.51E‐05
Human papillomavirus infection	297	5.05E‐05
mTOR signaling pathway	144	5.47E‐05
Proteoglycans in cancer	193	6.86E‐05
Synaptic vesicle cycle	63	7.90E‐05
Endocytosis	229	8.10E‐05
MAPK signaling pathway	272	9.50E‐05
Hippo signaling pathway	147	1.27E‐04
Rap1 signaling pathway	194	1.57E‐04
Fc gamma R‐mediated phagocytosis	88	1.64E‐04
Thyroid hormone signaling pathway	112	1.68E‐04
Signaling pathways regulating pluripotency of stem cells	133	1.86E‐04
Inositol phosphate metabolism	73	1.96E‐04
Glutamatergic synapse	110	2.13E‐04
PI3K‐Akt signaling pathway	320	2.36E‐04
Phagosome	141	2.41E‐04
HIF‐1 signaling pathway	97	2.52E‐04
Calcium signaling pathway	170	2.55E‐04
Platinum drug resistance	71	2.59E‐04
Breast cancer	140	2.68E‐04
AMPK signaling pathway	115	4.23E‐04
Epithelial cell signaling in Helicobacter pylori infection	67	4.48E‐04
Insulin resistance	103	4.82E‐04
HTLV‐I infection	235	4.86E‐04
Ras signaling pathway	217	5.13E‐04
Chemokine signaling pathway	172	5.18E‐04
Th17 cell differentiation	101	6.07E‐04
Osteoclast differentiation	120	7.57E‐04
Th1 and Th2 cell differentiation	87	8.47E‐04
Lysosome	118	9.35E‐04
Bacterial invasion of epithelial cells	74	9.89E‐04

### Verification of predicted differentially expressed miRNAs

**Figure 5:**
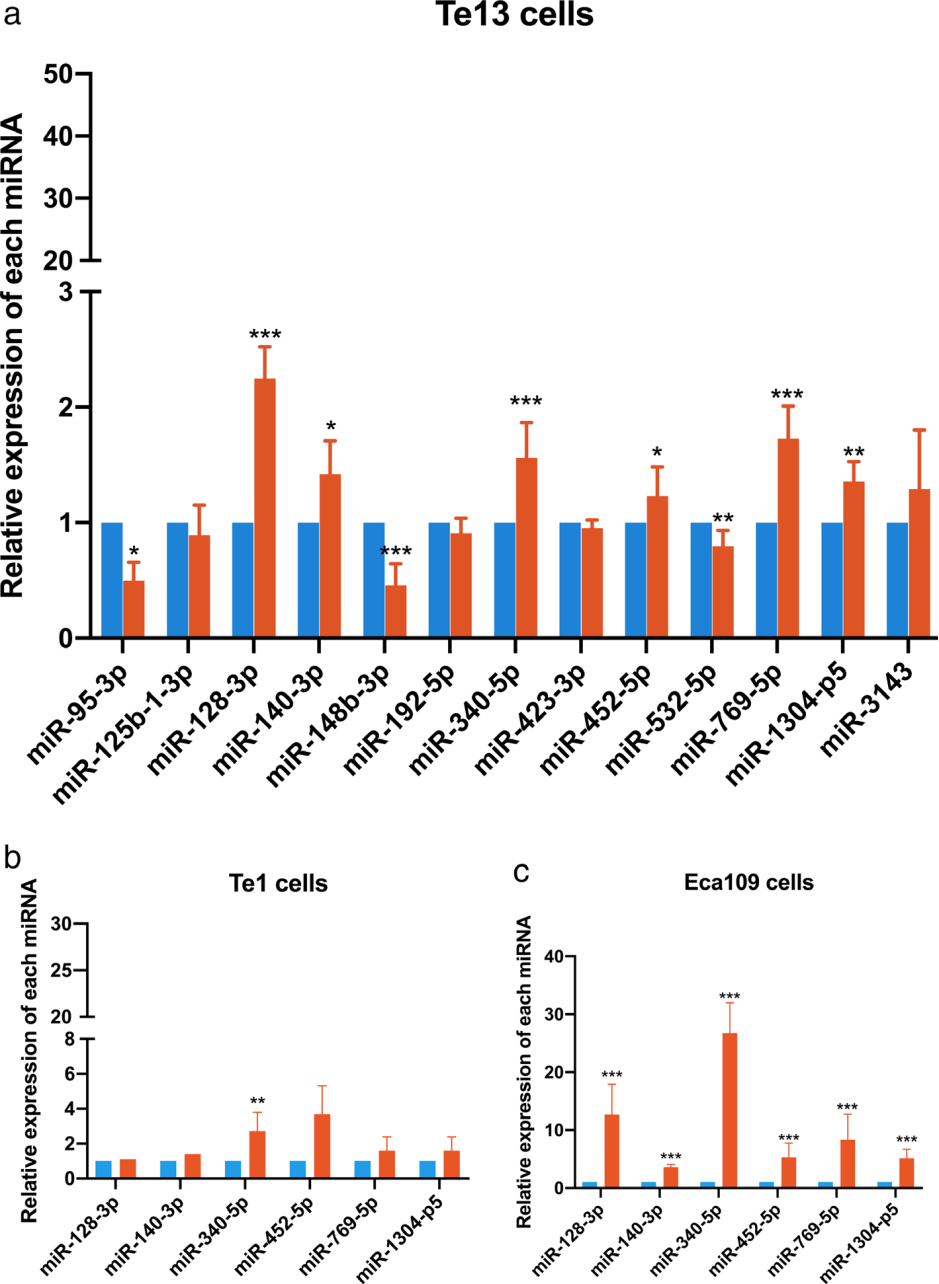
Verification of differentially expressed miRNA by RT‐PCR in (**a**) Te13 cells, (**b**) Te1cells and (**c**) ECA109 cells. 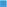 Normoxia, 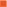 Hypoxia.

We selected 13 miRNAs which are upregulated in hypoxic EVs (*P* < 0.01) and have high or middle expression levels (hsa‐miR‐95‐3p, hsa‐miR‐128‐3p, hsa‐miR‐140‐3p, hsa‐miR‐148b‐3p, hsa‐miR‐192‐5p, hsa‐miR‐340‐5p, hsa‐miR‐125b‐1‐3p, hsa‐423‐3p, hsa‐miR‐452‐5p, hsa‐miR‐532‐5p, hsa‐miR‐769‐5p, hsa‐miR‐1304‐p5, hsa‐miR‐3143) and verified them in hypoxia EVs and normoxia EVs of Te13 cells by RT‐PCR. The results showed that miR‐128‐3p, miR‐140‐3p, miR‐340‐5p, miR‐452‐5p, miR‐769‐5p and miR‐1304‐p5 were significantly upregulated in hypoxic EVs (Fig.5(a)). We further verified these seven miRNAs in EVs of TE1 and ECA109 cells and found that miR‐340‐5p was overexpressed in hypoxic EVs of all three types of cells (Fig.5(b) and 5(c)).

## Discussion

Hypoxia, as a vital microenvironmental factor, plays a key role in the progression and metastasis of tumors. Tumor hypoxia leads to tumor characteristics such as resistance to apoptosis by alteration of oncogene expression, genomic instability, tumor angiogenesis, and epithelial to mesenchymal transition. Moreover, hypoxia is a major issue in the treatment of cancers.^9^, ^13^, ^14^ Destruction of tumor cells by ionizing radiation and certain drugs such as bleomycin is dependent on the presence of reactive oxygen species (ROS). During hypoxia, oxygen exhaustion results in decreased intracellular ROS and radioresistance and chemoresistance of tumors.^26^ However, DNA double‐stranded breaks caused by ionizing radiation are repaired by oxygen molecules since they have a higher affinity for unpaired electrons of free radicals.^6^ Based on recent research, HIF‐1 affects different aspects of immune response, including differentiation of immune cells and function of immune cells in the TME. Growing evidence suggests that antitumor immune response may be suppressed by tumor hypoxia.^27^

Here, for the first time, we report a basic study of miRNA profiles of EVs released from hypoxic ESCC cell lines. A set of exosomal miRNAs were selected based on the frequency or magnitude of variance in hypoxic versus normoxic cells. The TE‐13 cell line was cultured in a medium with EV‐depleted FBS to avoid bovine EV contamination. The cell line‐derived EVs were isolated by ultracentrifugation and washed with PBS to avoid protein aggregates and other carriers of extracellular miRNA contamination to the maximum extent. Using a sequencing approach, we obtained 10 810 miRNAs from the normoxic group and hypoxic group. Only 2601 miRNAs were detected in the normoxic group, and only 2990 miRNAs were detected in the hypoxic group and 5210 miRNAs were detected in both groups. Of the total 10 810 miRNAs, 50 were significantly upregulated and 34 were significantly downregulated after hypoxic treatment when compared to controls (*P* < 0.05).

Using these data, we performed a GO analysis and found that the biological function of the miRNAs was related to protein binding. It is reported that mammalian cells possess evolutionarily conserved endogenous mechanisms that enable them to respond to low‐oxygen conditions. This response is regulated by protein‐protein interactions and their downstream signaling pathways.^28^, ^29^ Given this biological function, it is likely that some key proteins might regulate the hypoxic responses. Hence, KEGG pathway analysis was performed,which showed that the differentially expressed miRNAs were mainly involved in the cancer‐related pathways and phospholipase D signaling pathway. The abnormal activation of tumorigenic signaling pathways is a comprehensive phenomenon in cancer and drives oncogenesis and malignant transformation. The key downstream proteins of these signaling pathways might interact with each other to form a feedback stimulation loop. Therefore, blockade of overactivated cancer pathways can be an important target for cancer therapy.^30^, ^31^ For instance, tyrosine kinase inhibitors have been successfully used to treat mutant EGFR non‐small cell lung cancer. Recent findings have shown that phosphatidic acid generated by phospholipase D play a role in numerous essential cellular functions, such as vesicular trafficking, exocytosis, autophagy, regulation of cellular metabolism, and tumorigenesis. Phospholipase D is a regulator of intercellular signaling and metabolic pathways, particularly in cells that are under stress.^32^, ^33^, ^34^ Cancer cells are characterized by the generation of lactic acid from glucose despite adequate oxygen for oxidative phosphorylation, known as the Warburg effect. The Warburg effect provides a strategy for cancer cells to survive under hypoxic conditions and nutrient deprivation. The shift from oxidative phosphorylation to anaerobic glycolysis in response to hypoxia is mediated by the production of HIF.^11^, ^35^ Early studies in renal and breast cancer cells have demonstrated that increased phospholipase D is required for the expression of HIF‐1 and HIF‐2, and aerobic glycolysis in these cells is dependent on elevated phospholipase D activity.^36^, ^37^, ^38^ In addition, some other pathways such as focal adhesion, autophagy and AGE‐RAGE with high enrichment may also be involved in hypoxic cancer biology. Focal adhesion pathway changes in cancer development and cancer adhesion have been reported to be the main factors for chemotherapy resistance,^39^ while autophagy has been widely reported to take part in multidrug resistance during cancer treatment.^40^ Moreover, a novel pathway which has been reported in cancer research in recent years is AGE‐RAGE pathway. Hypoxia‐induced glycolysis may lead to enhanced glucose uptake of tumor cells which results in accumulation of advanced glycation end products (AGE). The role of AGE in cancer progression is being extensively studied. In colorectal and oral cancer, silencing AGE‐RAGE signaling can repress cancer cells from proliferation and migration. Since tumor masses are innate hypoxia and highly glycated, inhibition of AGE‐RAGE interaction might be a potential therapeutic target.^41^ Our findings regarding the potential pathways involved in hypoxic EVs might provide new insight that hypoxic cancer cells can affect the surrounding TME in a paracrine manner.

Further verification experiment from RT‐PCR indicated that miR‐128‐3p, miR‐140‐3p, miR‐340‐5p, miR‐452‐5p, miR‐769‐5p and miR‐1304‐p5 were significantly upregulated in EVs from hypoxia TE‐13 cells while miR‐340‐5p was significantly upregulated in two other ESCC cells, ECA109 and TE‐1. These miRNAs even have inconsistent effect in cancer biology, such as miR‐340‐5p has been identified as both a tumor suppressor and tumor promoter in multicancers, including breast cancer, gastric cancer and non‐small cell lung cancer; thus, these validated miRNAs from RT‐PCR are worthy of further investigation in the future.

There are several limitations in this study. First, the sequencing data was based observations from ESCC cell lines. Considering tumor heterogeneity, our results might not include all the potential differentially expressed miRNAs in hypoxic ESCC cells. We did not validate all the obtained data in EVs from other ESCC cell lines due to the large number of altered miRNAs (50 upregulated and 34 downregulated). In conclusion, the miRNAs in EVs secreted by ESCC cells can undergo significant changes after hypoxia. The Warburg effect and shift from oxidative phosphorylation to anaerobic glycolysis in response to hypoxia may be associated with the intercellular communications via EVs in the hypoxic TME. More studies concerning mechanisms and phenotypic changes of phospholipase D activity, focal adhesion, autophagy, AGE‐RAGE and other pathways in the future will help to explore the relationship between hypoxic TME and EVs.

## Disclosure

The authors confirm there is no conflict of interest.

## Supporting information


**Table S1** Significantly altered miRNAs in hypoxic extracellular vesicles (*P* < 0.05).
**Table S2** KEGG pathways affected by altered exosomal miRNAs (*P* < 0.05)

## References

[1] Global Burden of Disease Cancer C , Fitzmaurice C , Dicker D *et al* The global burden of cancer 2013. JAMA Oncol 2015; 1 (4): 505–27. 26181261 10.1001/jamaoncol.2015.0735 PMC4500822

[2] Lagergren J , Smyth E , Cunningham D , Lagergren P . Oesophageal cancer. Lancet 2017; 390 (10110): 2383–96. 28648400 10.1016/S0140-6736(17)31462-9

[3] Zeng H , Zheng R , Guo Y *et al* Cancer survival in China, 2003‐2005: A population‐based study. Int J Cancer 2015; 136 (8): 1921–30. 25242378 10.1002/ijc.29227

[4] Gavin AT , Francisci S , Foschi R *et al* Oesophageal cancer survival in Europe: A EUROCARE‐4 study. Cancer Epidemiol 2012; 36 (6): 505–12. 22910036 10.1016/j.canep.2012.07.009

[5] Njei B , McCarty TR , Birk JW . Trends in esophageal cancer survival in United States adults from 1973 to 2009: A SEER database analysis. J Gastroenterol Hepatol 2016; 31 (6): 1141–6. 26749521 10.1111/jgh.13289 PMC4885788

[6] Horsman MR , Mortensen LS , Petersen JB , Busk M , Overgaard J . Imaging hypoxia to improve radiotherapy outcome. Nat Rev Clin Oncol 2012; 9 (12): 674–87. 23149893 10.1038/nrclinonc.2012.171

[7] Lyssiotis CA , Kimmelman AC . Metabolic interactions in the tumor microenvironment. Trends Cell Biol 2017; 27 (11): 863–75. 28734735 10.1016/j.tcb.2017.06.003 PMC5814137

[8] De Palma M , Biziato D , Petrova TV . Microenvironmental regulation of tumour angiogenesis. Nat Rev Cancer 2017; 17 (8): 457–74. 28706266 10.1038/nrc.2017.51

[9] Patel A , Sant S . Hypoxic tumor microenvironment: Opportunities to develop targeted therapies. Biotechnol Adv 2016; 34 (5): 803–12. 27143654 10.1016/j.biotechadv.2016.04.005 PMC4947437

[10] Maman S , Witz IP . A history of exploring cancer in context. Nat Rev Cancer 2018; 18 (6): 359–76. 29700396 10.1038/s41568-018-0006-7

[11] Cairns RA , Harris IS , Mak TW . Regulation of cancer cell metabolism. Nat Rev Cancer 2011; 11 (2): 85–95. 21258394 10.1038/nrc2981

[12] Dewhirst MW , Cao Y , Moeller B . Cycling hypoxia and free radicals regulate angiogenesis and radiotherapy response. Nat Rev Cancer 2008; 8 (6): 425–37. 18500244 10.1038/nrc2397 PMC3943205

[13] Erler JT , Cawthorne CJ , Williams KJ *et al* Hypoxia‐mediated down‐regulation of bid and bax in tumors occurs via hypoxia‐inducible factor 1‐dependent and ‐independent mechanisms and contributes to drug resistance. Mol Cell Biol 2004; 24 (7): 2875–89. 15024076 10.1128/MCB.24.7.2875-2889.2004 PMC371100

[14] Graeber TG , Osmanian C , Jacks T *et al* Hypoxia‐mediated selection of cells with diminished apoptotic potential in solid tumours. Nature 1996; 379 (6560): 88–91. 8538748 10.1038/379088a0

[15] Hill RP , Marie‐Egyptienne DT , Hedley DW . Cancer stem cells, hypoxia and metastasis. Semin Radiat Oncol 2009; 19 (2): 106–11. 19249648 10.1016/j.semradonc.2008.12.002

[16] Kioi M , Vogel H , Schultz G , Hoffman RM , Harsh GR , Brown JM . Inhibition of vasculogenesis, but not angiogenesis, prevents the recurrence of glioblastoma after irradiation in mice. J Clin Invest 2010; 120 (3): 694–705. 20179352 10.1172/JCI40283 PMC2827954

[17] Yang X , Li Y , Zou L , Zhu Z . Role of exosomes in crosstalk between cancer‐associated fibroblasts and cancer cells. Front Oncol 2019; 9: 356. 31131261 10.3389/fonc.2019.00356 PMC6510008

[18] Shao C , Yang F , Miao S *et al* Role of hypoxia‐induced exosomes in tumor biology. Mol Cancer 2018; 17 (1): 120. 30098600 10.1186/s12943-018-0869-y PMC6087002

[19] Rupaimoole R , Slack FJ . MicroRNA therapeutics: Towards a new era for the management of cancer and other diseases. Nat Rev Drug Discov 2017; 16 (3): 203–22. 28209991 10.1038/nrd.2016.246

[20] Xu R , Rai A , Chen M , Suwakulsiri W , Greening DW , Simpson RJ . Extracellular vesicles in cancer ‐ implications for future improvements in cancer care. Nat Rev Clin Oncol 2018; 15 (10): 617–38. 29795272 10.1038/s41571-018-0036-9

[21] Fu M , Gu J , Jiang P , Qian H , Xu W , Zhang X . Exosomes in gastric cancer: Roles, mechanisms, and applications. Mol Cancer 2019; 18 (1): 41. 30876419 10.1186/s12943-019-1001-7 PMC6419325

[22] He C , Zheng S , Luo Y , Wang B . Exosome theranostics: Biology and translational medicine. Theranostics 2018; 8 (1): 237–55. 29290805 10.7150/thno.21945 PMC5743472

[23] Joyce DP , Kerin MJ , Dwyer RM . Exosome‐encapsulated microRNAs as circulating biomarkers for breast cancer. Int J Cancer 2016; 139 (7): 1443–8. 27170104 10.1002/ijc.30179

[24] Tang MK , Wong AS . Exosomes: Emerging biomarkers and targets for ovarian cancer. Cancer Lett 2015; 367 (1): 26–33. 26189430 10.1016/j.canlet.2015.07.014

[25] Zhou L , Lv T , Zhang Q *et al* The biology, function and clinical implications of exosomes in lung cancer. Cancer Lett 2017; 407: 84–92. 28807820 10.1016/j.canlet.2017.08.003

[26] Rey S , Schito L , Koritzinsky M , Wouters BG . Molecular targeting of hypoxia in radiotherapy. Adv Drug Deliv Rev 2017; 109: 45–62. 27771366 10.1016/j.addr.2016.10.002

[27] Wilson WR , Hay MP . Targeting hypoxia in cancer therapy. Nat Rev Cancer 2011; 11 (6): 393–410. 21606941 10.1038/nrc3064

[28] Majmundar AJ , Wong WJ , Simon MC . Hypoxia‐inducible factors and the response to hypoxic stress. Mol Cell 2010; 40 (2): 294–309. 20965423 10.1016/j.molcel.2010.09.022 PMC3143508

[29] Shah YM , Xie L . Hypoxia‐inducible factors link iron homeostasis and erythropoiesis. Gastroenterology 2014; 146 (3): 630–42. 24389303 10.1053/j.gastro.2013.12.031 PMC3943938

[30] Kolch W , Halasz M , Granovskaya M , Kholodenko BN . The dynamic control of signal transduction networks in cancer cells. Nat Rev Cancer 2015; 15 (9): 515–27. 26289315 10.1038/nrc3983

[31] Mullen PJ , Yu R , Longo J , Archer MC , Penn LZ . The interplay between cell signalling and the mevalonate pathway in cancer. Nat Rev Cancer 2016; 16 (11): 718–31. 27562463 10.1038/nrc.2016.76

[32] Brown HA , Thomas PG , Lindsley CW . Targeting phospholipase D in cancer, infection and neurodegenerative disorders. Nat Rev Drug Discov 2017; 16 (5): 351–67. 28209987 10.1038/nrd.2016.252 PMC6040825

[33] Bruntz RC , Lindsley CW , Brown HA . Phospholipase D signaling pathways and phosphatidic acid as therapeutic targets in cancer. Pharmacol Rev 2014; 66 (4): 1033–79. 25244928 10.1124/pr.114.009217 PMC4180337

[34] Frohman MA . The phospholipase D superfamily as therapeutic targets. Trends Pharmacol Sci 2015; 36 (3): 137–44. 25661257 10.1016/j.tips.2015.01.001 PMC4355084

[35] Vander Heiden MG , Cantley LC , Thompson CB . Understanding the Warburg effect: The metabolic requirements of cell proliferation. Science 2009; 324 (5930): 1029–33. 19460998 10.1126/science.1160809 PMC2849637

[36] Toschi A , Edelstein J , Rockwell P , Ohh M , Foster DA . HIF alpha expression in VHL‐deficient renal cancer cells is dependent on phospholipase D. Oncogene 2008; 27 (19): 2746–53. 17998935 10.1038/sj.onc.1210927

[37] Toschi A , Lee E , Thompson S *et al* Phospholipase D‐mTOR requirement for the Warburg effect in human cancer cells. Cancer Lett 2010; 299 (1): 72–9. 20805015 10.1016/j.canlet.2010.08.006 PMC3010225

[38] Zheng Y , Rodrik V , Toschi A *et al* Phospholipase D couples survival and migration signals in stress response of human cancer cells. J Biol Chem 2006; 281 (23): 15862–8. 16595654 10.1074/jbc.M600660200

[39] Eke I , Cordes N . Focal adhesion signaling and therapy resistance in cancer. Semin Cancer Biol 2015; 31: 65–75. 25117005 10.1016/j.semcancer.2014.07.009

[40] Li YJ , Lei YH , Yao N *et al* Autophagy and multidrug resistance in cancer. Chin J Cancer 2017; 36 (1): 52. 28646911 10.1186/s40880-017-0219-2 PMC5482965

[41] Khan MI , Rath S , Adhami VM , Mukhtar H . Hypoxia driven glycation: Mechanisms and therapeutic opportunities. Semin Cancer Biol 2018; 49: 75–82. 28546110 10.1016/j.semcancer.2017.05.008 PMC5699980

