# Supplementary material for: Hypoxia induced changes in miRNAs and their target mRNAs in extracellular vesicles of esophageal squamous cancer cells
Source: Thorac Cancer. 2020 Jan 10;11(3):570–80. doi: 10.1111/1759-7714.13295 (PMC7049507; doi:10.1111/1759-7714.13295)
Supplement: Supplementary file 1 — Table S1 Significantly altered miRNAs in hypoxic extracellular vesicles (P < 0.05). Table S2 KEGG pathways affected by altered exosomal miRNAs (P < 0.05) [file TCA-11-570-s001.docx]

**Supporting information:**

**Table S1**: Significantly altered miRNAs in hypoxic extracellular vesicles (p<0.05)

| **miRNA** | **miRNA_Seq** | **Regulation** | **Fold Change** | p value |
| --- | --- | --- | --- | --- |
| hsa-miR-148b-3p | TCAGTGCATCACAGAACTTTGT | up | 2.64 | 0.000328863 |
| hsa-miR-1262 | ATGGGTGAATTTGTAGAAGGAT | up | inf | 0.000869564 |
| hsa-miR-423-3p | AGCTCGGTCTGAGGCCCCTCAGT | up | 1.45 | 0.001446868 |
| hsa-miR-769-5p | TGAGACCTCTGGGTTCTGAGCT | up | 3.06 | 0.002934105 |
| hsa-mir-1304-p5 | CGGTTTGAGGCTACAGTGAGAT | up | 3.71 | 0.00333358 |
| PC-3p-73334_38 | CTTGAGGAAGGCTGTCCCT | down | 0.23 | 0.003489675 |
| hsa-miR-128-3p | TCACAGTGAACCGGTCTCTTT | up | 2.02 | 0.003914834 |
| PC-3p-2356_1595 | GTCGCTGGGTGTAGACCC | down | 0.17 | 0.004213607 |
| hsa-miR-106b-5p | TAAAGTGCTGACAGTGCAGAT | down | 0.69 | 0.004547923 |
| hsa-miR-369-3p | AATAATACATGGTTGATCTTT | down | 0.39 | 0.005009377 |
| mmu-let-7j_1ss8TG | TGAGGTAGTAGTTTGTGCTGTTAT | up | 4.47 | 0.005458252 |
| hsa-miR-95-3p_R-1 | TTCAACGGGTATTTATTGAGC | up | 2.15 | 0.0062081 |
| hsa-miR-532-5p | CATGCCTTGAGTGTAGGACCGT | up | 2.11 | 0.006414664 |
| hsa-miR-140-3p_L-1R+2 | ACCACAGGGTAGAACCACGGAC | up | 2.21 | 0.006570997 |
| PC-3p-200028_13 | AATCCCTTACCATCGGCCA | down | 0.12 | 0.006795539 |
| hsa-miR-192-5p | CTGACCTATGAATTGACAGCC | up | 2.15 | 0.006911668 |
| hsa-miR-3143_R-3 | ATAACATTGTAAAGCGCTTCTT | up | inf | 0.006983654 |
| hsa-miR-574-3p | CACGCTCATGCACACACCCACA | down | 0.60 | 0.007520111 |
| hsa-miR-340-5p | TTATAAAGCAATGAGACTGATT | up | 2.01 | 0.007744756 |
| PC-5p-151105_17 | CTGAAACCGTTTGCTTAC | down | -inf | 0.008038862 |
| hsa-miR-409-3p | GAATGTTGCTCGGTGAACCCCT | down | 0.39 | 0.008233985 |
| hsa-miR-452-5p_L-3R+3 | TGTTTGCAGAGGAAACTGAGAC | up | 2.31 | 0.008441793 |
| hsa-miR-125b-1-3p_R-1_1ss21CT | ACGGGTTAGGCTCTTGGGAGT | up | 9.15 | 0.00968456 |
| hsa-miR-144-3p | TACAGTATAGATGATGTACT | down | 0.28 | 0.009919166 |
| hsa-miR-4804-5p_L+1_1ss7CG | TTTGGAGGGTAAGGTTAAGCAA | up | inf | 0.010424628 |
| mmr-miR-1839_L+1 | AAGGTAGATAGAACAGGTCTTGT | up | 2.74 | 0.012924266 |
| hsa-miR-651-5p_R-1 | TTTAGGATAAGCTTGACTTTT | up | 3.49 | 0.013829607 |
| PC-5p-33017_111 | TGGCTGGGGCGGCACATC | down | 0.28 | 0.013923959 |
| mmu-miR-5106_L-5_1ss7GT | TTTAGCTCAGTTGGCAGA | down | 0.23 | 0.014444777 |
| hsa-miR-5010-5p | AGGGGGATGGCAGAGCAAAATT | up | inf | 0.014810814 |
| PC-5p-69635_41 | AGCCTGGAAGCTGGAGCCTGCAG | up | inf | 0.015375722 |
| hsa-miR-548bc_L+1 | CAAAAACTGTGATTACTTTTGC | up | 6.48 | 0.015489606 |
| hsa-miR-217-5p | TACTGCATCAGGAACTGATTGGA | down | 0.10 | 0.015682558 |
| bta-miR-2285k_R-1 | AAAACCGGAATGAACTTTTT | down | -inf | 0.015764234 |
| PC-3p-98279_26 | GTCCCGTCTCTCGCGCCA | down | 0.09 | 0.017493133 |
| hsa-let-7a-3p_R+1 | CTATACAATCTACTGTCTTTCT | up | 2.58 | 0.018830902 |
| hsa-miR-450b-5p_R-1 | TTTTGCAATATGTTCCTGAAT | up | 2.22 | 0.018942492 |
| hsa-let-7a-3p_R+1_1ss8AG | CTATACAGTCTACTGTCTTTCT | up | 2.53 | 0.019486839 |
| hsa-let-7f-2-3p_1ss22CT | CTATACAGTCTACTGTCTTTCT | up | 2.53 | 0.019486839 |
| hsa-miR-767-5p_R-1 | TGCACCATGGTTGTCTGAGCAT | up | 2.65 | 0.020550091 |
| hsa-mir-548p-p3 | CAAAAACTGCAGTTACTTTTGC | up | 7.43 | 0.020624175 |
| hsa-miR-548av-3p_L+2_1 | CAAAAACTGCAGTTACTTTTGC | up | 7.43 | 0.020624175 |
| hsa-miR-548av-3p_L+2_2 | CCAAAACTGCAGTTACTTTTGC | up | 7.43 | 0.020624175 |
| eca-mir-8986a-p5_1ss1GA | ATCGAGGCTAGAGTCACGCTTG | up | inf | 0.021515591 |
| hsa-miR-675-3p_R+2 | CTGTATGCCCTCACCGCTCAGC | up | 1.78 | 0.021975813 |
| hsa-miR-941 | CACCCGGCTGTGTGCACATGTGC | up | 1.74 | 0.022470307 |
| hsa-miR-27b-3p | TTCACAGTGGCTAAGTTCTGC | up | 2.28 | 0.023186219 |
| hsa-miR-7-5p_R-1 | TGGAAGACTAGTGATTTTGTTGT | up | 1.61 | 0.02322044 |
| PC-5p-42571_80 | AGTGTGGAAGAGATAACCG | down | 0.23 | 0.024197888 |
| hsa-miR-579-5p_R-1 | TCGCGGTTTGTGCCAGATGAC | up | inf | 0.025081989 |
| hsa-miR-34a-5p | TGGCAGTGTCTTAGCTGGTTGT | down | 0.60 | 0.025337943 |
| PC-3p-14721_282 | GAGATTCCGAAAGTAGTG | down | 0.19 | 0.025845657 |
| hsa-miR-30a-3p | CTTTCAGTCGGATGTTTGCAGC | up | 1.51 | 0.025891854 |
| hsa-miR-99b-3p_R-1 | CAAGCTCGTGTCTGTGGGTCC | up | 2.06 | 0.026076101 |
| hsa-miR-208b-3p_L+1R-2 | TATAAGACGAACAAAAGGTTT | down | 0.60 | 0.026661606 |
| mmu-mir-6236-p5_1 | AAATGGATGGCGCTGGAG | down | 0.46 | 0.026696834 |
| mmu-mir-6236-p5_2 | ATCAACTAGCCCTGAAAAT | down | 0.46 | 0.026696834 |
| hsa-miR-1283_L-1 | CTACAAAGGAAAGCGCTTTCT | up | 2.20 | 0.027371719 |
| hsa-miR-625-3p_R-1 | GACTATAGAACTTTCCCCCTC | down | 0.26 | 0.027897252 |
| hsa-let-7c-5p | TGAGGTAGTAGGTTGTATGGTT | up | 2.71 | 0.029471787 |
| bta-miR-6119-5p_R-2 | AGAGGTAAAAAATTGATTTGA | down | 0.64 | 0.029728728 |
| hsa-miR-22-5p_R-1 | AGTTCTTCAGTGGCAAGCTTT | down | 0.52 | 0.0297875 |
| hsa-miR-629-5p_R+1 | TGGGTTTACGTTGGGAGAACTT | up | 1.63 | 0.030832835 |
| hsa-let-7f-5p | TGAGGTAGTAGATTGTATAGTT | up | 1.97 | 0.032270344 |
| hsa-miR-30d-3p | CTTTCAGTCAGATGTTTGCTGC | up | 5.24 | 0.033389982 |
| PC-5p-12960_321 | AGCGGTACGTGAGTTGGG | down | 0.32 | 0.033541351 |
| hsa-miR-151a-3p | CTAGACTGAAGCTCCTTGAGG | up | 1.64 | 0.03365824 |
| hsa-miR-142-5p_L+2R-3 | CCCATAAAGTAGAAAGCACT | down | 0.61 | 0.034692204 |
| hsa-miR-589-5p_R-1 | TGAGAACCACGTCTGCTCTGA | up | 2.22 | 0.034693013 |
| PC-3p-126478_20 | AGACTCTTCTCCCGCTCCA | down | -inf | 0.035403881 |
| hsa-miR-92a-3p | TATTGCACTTGTCCCGGCCTGT | up | 1.79 | 0.037500941 |
| hsa-miR-4482-3p_R+1 | TTTCTATTTCTCAGTGGGGCTCT | up | inf | 0.03821187 |
| hsa-miR-9-5p | TCTTTGGTTATCTAGCTGTATGA | down | 0.23 | 0.039632993 |
| hsa-miR-185-5p | TGGAGAGAAAGGCAGTTCCTGA | up | 2.41 | 0.040755674 |
| PC-3p-32762_112 | TGCTTCCAGGAAAAGCCAC | down | 0.11 | 0.040809803 |
| hsa-miR-1323 | TCAAAACTGAGGGGCATTTTCT | up | inf | 0.042318068 |
| hsa-let-7i-5p | TGAGGTAGTAGTTTGTGCTGTT | up | 2.51 | 0.043275458 |
| mmu-miR-5106_L-5_1ss20CT | TGTAGCTCAGTTGGTAGA | down | 0.20 | 0.043708328 |
| PC-3p-11088_376 | GCAGGTCCCAAGGGTATG | down | 0.20 | 0.044118421 |
| mmu-mir-5119-p5_1ss1GC | CTCATCTCATCCTGGGGC | down | 0.11 | 0.04430032 |
| PC-3p-113010_22 | CATGGCGCCAGCTGTGCAGC | down | 0.22 | 0.044436327 |
| PC-5p-46762_70 | ATCCTTCCTCCCCAGCCA | down | 0.09 | 0.044545911 |
| hsa-let-7g-5p | TGAGGTAGTAGTTTGTACAGTT | up | 1.46 | 0.047386886 |
| hsa-mir-7110-p3_1ss19AC | TCTCTCTCTCTCTCTCCCC | down | 0.02 | 0.049554782 |

**Table S2**: KEGG pathways affected by altered exosomal miRNAs (p<0.05)

| **Pathway ID** | **Pathway description** | ***p* value** |
| --- | --- | --- |
| ko05200 | Pathways in cancer | 1.02512E-07 |
| ko04072 | Phospholipase D signaling pathway | 4.87835E-06 |
| ko04933 | AGE-RAGE signaling pathway in diabetic complications | 5.9019E-06 |
| ko04140 | Autophagy - animal | 7.36107E-06 |
| ko04510 | Focal adhesion | 1.16162E-05 |
| ko04611 | Platelet activation | 1.59293E-05 |
| ko04068 | FoxO signaling pathway | 2.76288E-05 |
| ko04360 | Axon guidance | 4.50821E-05 |
| ko05165 | Human papillomavirus infection | 5.04753E-05 |
| ko04150 | mTOR signaling pathway | 5.46691E-05 |
| ko05205 | Proteoglycans in cancer | 6.86396E-05 |
| ko04721 | Synaptic vesicle cycle | 7.89712E-05 |
| ko04144 | Endocytosis | 8.10233E-05 |
| ko04010 | MAPK signaling pathway | 9.50307E-05 |
| ko04390 | Hippo signaling pathway | 0.000126891 |
| ko04015 | Rap1 signaling pathway | 0.000157441 |
| ko04666 | Fc gamma R-mediated phagocytosis | 0.00016406 |
| ko04919 | Thyroid hormone signaling pathway | 0.000168213 |
| ko04550 | Signaling pathways regulating pluripotency of stem cells | 0.000186422 |
| ko00562 | Inositol phosphate metabolism | 0.000196274 |
| ko04724 | Glutamatergic synapse | 0.000213035 |
| ko04151 | PI3K-Akt signaling pathway | 0.000236212 |
| ko04145 | Phagosome | 0.000240764 |
| ko04066 | HIF-1 signaling pathway | 0.000252183 |
| ko04020 | Calcium signaling pathway | 0.000255479 |
| ko01524 | Platinum drug resistance | 0.000258706 |
| ko05224 | Breast cancer | 0.000267598 |
| ko04152 | AMPK signaling pathway | 0.000422605 |
| ko05120 | Epithelial cell signaling in Helicobacter pylori infection | 0.000448446 |
| ko04931 | Insulin resistance | 0.000482333 |
| ko05166 | HTLV-I infection | 0.000485698 |
| ko04014 | Ras signaling pathway | 0.000512666 |
| ko04062 | Chemokine signaling pathway | 0.000517654 |
| ko04659 | Th17 cell differentiation | 0.000607412 |
| ko04380 | Osteoclast differentiation | 0.000757163 |
| ko04658 | Th1 and Th2 cell differentiation | 0.000847489 |
| ko04142 | Lysosome | 0.000934522 |
| ko05100 | Bacterial invasion of epithelial cells | 0.000988995 |
| ko04213 | Longevity regulating pathway - multiple species | 0.001017084 |
| ko04930 | Type II diabetes mellitus | 0.001019307 |
| ko04070 | Phosphatidylinositol signaling system | 0.001074425 |
| ko05231 | Choline metabolism in cancer | 0.00120293 |
| ko04012 | ErbB signaling pathway | 0.001363554 |
| ko03320 | PPAR signaling pathway | 0.001634874 |
| ko05412 | Arrhythmogenic right ventricular cardiomyopathy (ARVC) | 0.001634874 |
| ko04071 | Sphingolipid signaling pathway | 0.001741184 |
| ko01522 | Endocrine resistance | 0.001882885 |
| ko04910 | Insulin signaling pathway | 0.001950515 |
| ko05418 | Fluid shear stress and atherosclerosis | 0.001950515 |
| ko04210 | Apoptosis | 0.001950515 |
| ko05226 | Gastric cancer | 0.001998321 |
| ko05225 | Hepatocellular carcinoma | 0.002008957 |
| ko04218 | Cellular senescence | 0.002015997 |
| ko05169 | Epstein-Barr virus infection | 0.002045973 |
| ko04917 | Prolactin signaling pathway | 0.002097684 |
| ko04371 | Apelin signaling pathway | 0.002145287 |
| ko04120 | Ubiquitin mediated proteolysis | 0.002145287 |
| ko04512 | ECM-receptor interaction | 0.002180883 |
| ko05211 | Renal cell carcinoma | 0.002374833 |
| ko04920 | Adipocytokine signaling pathway | 0.002374833 |
| ko04141 | Protein processing in endoplasmic reticulum | 0.002609083 |
| ko04660 | T cell receptor signaling pathway | 0.002665304 |
| ko01521 | EGFR tyrosine kinase inhibitor resistance | 0.003088731 |
| ko05142 | Chagas disease (American trypanosomiasis) | 0.003285008 |
| ko05131 | Shigellosis | 0.003886395 |
| ko05014 | Amyotrophic lateral sclerosis (ALS) | 0.004458753 |
| ko05212 | Pancreatic cancer | 0.004882976 |
| ko05215 | Prostate cancer | 0.004963137 |
| ko04350 | TGF-beta signaling pathway | 0.006233025 |
| ko04810 | Regulation of actin cytoskeleton | 0.006479692 |
| ko04330 | Notch signaling pathway | 0.006629767 |
| ko04520 | Adherens junction | 0.006850833 |
| ko04730 | Long-term depression | 0.007126431 |
| ko04310 | Wnt signaling pathway | 0.007422126 |
| ko04961 | Endocrine and other factor-regulated calcium reabsorption | 0.007561684 |
| ko05140 | Leishmaniasis | 0.007661858 |
| ko04146 | Peroxisome | 0.007701071 |
| ko00310 | Lysine degradation | 0.008034237 |
| ko04530 | Tight junction | 0.008252749 |
| ko04662 | B cell receptor signaling pathway | 0.008564496 |
| ko03018 | RNA degradation | 0.010534612 |
| ko04211 | Longevity regulating pathway - mammal | 0.011065086 |
| ko05220 | Chronic myeloid leukemia | 0.011681503 |
| ko04540 | Gap junction | 0.012202313 |
| ko04022 | cGMP - PKG signaling pathway | 0.013979785 |
| ko04921 | Oxytocin signaling pathway | 0.016053251 |
| ko05410 | Hypertrophic cardiomyopathy (HCM) | 0.01630854 |
| ko04924 | Renin secretion | 0.016516167 |
| ko05321 | Inflammatiory bowel disease (IBD) | 0.01838879 |
| ko00380 | Tryptophan metabolism | 0.018773051 |
| ko05222 | Small cell lung cancer | 0.01956968 |
| ko04725 | Cholinergic synapse | 0.020016152 |
| ko05145 | Toxoplasmosis | 0.020016152 |
| ko04726 | Serotonergic synapse | 0.020016152 |
| ko04950 | Maturity onset diabetes of the young | 0.020490947 |
| ko05032 | Morphine addiction | 0.021410413 |
| ko05210 | Colorectal cancer | 0.02144928 |
| ko05110 | Vibrio cholerae infection | 0.02308437 |
| ko00062 | Fatty acid elongation | 0.023802516 |
| ko04722 | Neurotrophin signaling pathway | 0.024245041 |
| ko00534 | Glycosaminoglycan biosynthesis - heparan sulfate / heparin | 0.027648654 |
| ko05414 | Dilated cardiomyopathy (DCM) | 0.027933964 |
| ko00280 | Valine, leucine and isoleucine degradation | 0.028995764 |
| ko04727 | GABAergic synapse | 0.030484561 |
| ko04115 | p53 signaling pathway | 0.031754471 |
| ko00564 | Glycerophospholipid metabolism | 0.031760338 |
| ko04750 | Inflammatory mediator regulation of TRP channels | 0.031760338 |
| ko01040 | Biosynthesis of unsaturated fatty acids | 0.032115555 |
| ko04340 | Hedgehog signaling pathway | 0.032464006 |
| ko04514 | Cell adhesion molecules (CAMs) | 0.033067228 |
| ko04713 | Circadian entrainment | 0.034495844 |
| ko04914 | Progesterone-mediated oocyte maturation | 0.034495844 |
| ko05340 | Primary immunodeficiency | 0.035428308 |
| ko05323 | Rheumatoid arthritis | 0.03623358 |
| ko04270 | Vascular smooth muscle contraction | 0.038349211 |
| ko04728 | Dopaminergic synapse | 0.038349211 |
| ko05223 | Non-small cell lung cancer | 0.038474722 |
| ko00270 | Cysteine and methionine metabolism | 0.04060723 |
| ko04064 | NF-kappa B signaling pathway | 0.040614335 |
| ko05130 | Pathogenic Escherichia coli infection | 0.042387982 |
| ko00071 | Fatty acid degradation | 0.045364315 |
| ko04024 | cAMP signaling pathway | 0.04642418 |
| ko03460 | Fanconi anemia pathway | 0.046896868 |
